# Association of Qualified Clinical Data Registry Clinician Dashboard Engagement With Performance on Quality-of-Care Measures: Cross-Sectional Analysis

**DOI:** 10.2196/72709

**Published:** 2025-09-04

**Authors:** Emma Kersey, Jing Li, Julia Adler-Milstein, Jinoos Yazdany, Stephen Shiboski, Gabriela Schmajuk

**Affiliations:** 1 Division of Rheumatology, Department of Medicine, University of California, San Francisco, San Francisco, CA, United States; 2 Division of Clinical Informatics and Digital Transformation, Department of Medicine, University of California, San Francisco, CA, United States; 3 Rheumatology Section, Department of Medicine, San Francisco VA Medical Center, 4150 Clement St, Mailstop 111R, San Francisco, CA, 94027, United States, 1 4157502104

**Keywords:** Qualified Clinical Data Registries, web-based dashboard, engagement, quality of care, rheumatology

## Abstract

**Background:**

Qualified Clinical Data Registries (QCDRs) have proliferated across many medical specialties, facilitating quality measure performance monitoring and reporting in programs like the CMS Merit-based Incentive Payment System. Many of these QCDRs offer web-based, clinician-facing dashboards to support quality improvement. However, it is unknown whether engagement with such dashboards is associated with improvements in quality of care.

**Objective:**

We investigated the cross-sectional relationship between engagement with a QCDR dashboard and quality measure performance.

**Methods:**

Data derived from a rheumatology QCDR (“Rheumatology Informatics System for Effectiveness [RISE]”) and audit log data from the dashboard (exposure) and Merit-based Incentive Payment System submission data (outcome) from 2020‐2022 were included. Among practices participating in RISE, we assessed aggregated engagement with the QCDR dashboard and quality performance for 8 rheumatology-specific measures at the practice level. For each measure, the binomial generalized linear model was used to examine the association between dashboard engagement and measure performance, adjusting for EHR vendor, study year, and clustering at the practice level to account for repeated measures. Two types of engagement were analyzed: (1) measure-specific (interactions with patient-level information for a particular measure) and (2) global (interactions with any feature of the dashboard, classified into 4 profiles). Linear trends between the level of dashboard engagement and performance were also tested in the global analysis.

**Results:**

In total, 211 practices were included in the study; over half were single-specialty practices. During their first year in the study, 65% of the practices had “most” or “moderate” levels of global engagement. In measure-specific analyses, we observed a positive but nonsignificant association of each individual and “any” actions with performance on 6‐8 measures. However, having a ≥90th percentile number of drill-down views on 1 measure (rheumatoid arthritis (RA) periodic disease activity assessment) was statistically significant (odds ratio [OR] 2.3, 95% CI 1.2‐4.3). In global analyses, we observed a similar pattern, where practices “most” engaged with the dashboard had higher odds of better performance compared to those with “none.” In total, 4 measures (osteoporosis screening, RA functional status assessment, RA periodic disease activity assessment, and gout serum urate target) had a statistically significant association with engagement and exhibited a “dose-response” relationship (*P*=.004, .02, <.001, and .04, respectively, for trend). Practices with “any” global engagement had higher performance on 6 out of 8 measures, again, with RA periodic disease activity assessment being statistically significant (OR 2.9, 95% CI 1.3‐6.6).

**Conclusions:**

We found that higher levels of engagement were associated with higher performance on some, but not all, rheumatology-specific quality measures. Additional work is needed to understand whether the dashboard facilitates quality improvement or is merely a marker for high-performing practices.

## Introduction

Over the past 2 decades, electronic health records (EHRs) have transformed the way clinical data are captured, stored, and used in health care delivery. This digital transformation has enabled the development of large-scale quality reporting infrastructures, including Qualified Clinical Data Registries (QCDRs). QCDRs play a central role in modern value-based care initiatives by extracting structured EHR data to support quality measurement and reporting in programs such as the Centers for Medicare and Medicaid Services (CMS) Merit-based Incentive Payment System (MIPS).

To facilitate quality improvement and compliance with reporting requirements, many QCDRs have implemented web-based, clinician-facing dashboards. These dashboards allow providers to track their performance on quality measures in near-real-time, offering a proactive tool to identify care gaps before annual reporting deadlines. In addition to aggregate performance summaries, many dashboards include patient-level drill-downs that enable clinicians to identify individuals not meeting specific quality measures, thereby supporting targeted interventions [1-5].

Despite their increasing use, the actual impact of QCDR dashboards on clinical performance remains unclear. Most prior studies have evaluated the effectiveness of dashboards by comparing performance before and after implementation [6-16]. These studies often report mixed results and rarely account for how clinicians engage with the dashboard over time. Furthermore, when dashboard engagement has been analyzed, it is typically measured using 1-dimensional indicators such as login counts or page views, which may fail to capture the complexity of how these tools are integrated into clinical workflows [16-19].

To address these limitations, we conducted a study to examine whether more meaningful engagement with a QCDR dashboard was associated with improved performance on quality measures. We focused on the Rheumatology Informatics System for Effectiveness (RISE), a national QCDR developed by the American College of Rheumatology (ACR) and quality measures relevant to rheumatology care. To capture the multifaceted nature of engagement, we applied the breadth-depth-context (BDC) framework—a structured approach that considers not only how often clinicians use the dashboard, but also the range and specificity of their interactions [20]. We then evaluated whether BDC-defined engagement profiles, as well as actions related to specific measures, were associated with performance on 8 rheumatology-specific quality measures. We hypothesized that higher levels of dashboard engagement would be associated with better performance on these quality measures.

## Methods

### Data Sources

The first source of data was that derived from quality measure performance. The RISE registry is a national EHR-enabled QCDR that continuously extracts data collected during clinical care from EHRs of participating practices [21]. Data are centrally aggregated, and quality measure performance is calculated by the registry vendor for selected Quality Payment Program (QPP) measures and ACR-stewarded QCDR measures.

The second source of data was the RISE dashboard and associated audit log data. Quality measure performance data are displayed on a web-based dashboard designed to facilitate quality improvement activities and submission of quality measure performance information to CMS by practice personnel. The RISE QCDR dashboard is available to personnel from participating practices. Features of the dashboard include practice- and clinician-level performance for a series of quality measures; overall performance and registry benchmarks are visible on the landing page.

Practice personnel can interact with the dashboard using additional actions, such as “drill-down views” to view patient-level data on the selected measures or “drill-down exports” to download patient-level data for offline access (see Appendix A in Multimedia Appendix 1).

They can generate performance summary reports for selected quality measures or across multiple measures.

Dashboard audit log files contain records of every session and action performed on the RISE dashboard. Data available for user sessions included session date and any executed dashboard actions (such as drill-down views or exports) applied to a specific quality measure (if any).

The third source of data was the study population and the study period*.* Included practices were required to have (1) at least 1 individual from that practice with an active RISE dashboard account for a minimum of 6 months during at least 1 study year and (2) available quality measure performance data calculated by the vendor for the same years. The study period was divided into 3 study years: 2020, 2021, and 2022. Included practices had at least 1 quality measure performance reported to CMS during at least 1 study year. Practices could be included more than once in the analysis for a particular measure if they met the inclusion criteria during more than 1 study year.

The outcome was quality measure performance. We examined performance on 8 rheumatology-specific quality measures at the practice level for each calendar year (2020‐2022). We included these measures because they align with care routinely provided by rheumatologists: QPP39 (osteoporosis screening), QPP178 (rheumatoid arthritis [RA] functional status assessment), QPP177 (RA periodic disease activity assessment), QPP176 (tuberculosis [TB] safety screening), ACR14 (gout serum urate (SU) target), ACR10 (hepatitis B virus [HBV] safety screening), ACR15 (safe hydroxychloroquine [HCQ] dosing), and ACR12 (psoriatic arthritis [PsA] disease activity assessment); see complete measure descriptions in the Practice and Quality Measure Characteristics section and Appendix B in Multimedia Appendix 1. In order to be included in the analysis for a particular measure, a practice was required to have at least 20 patients eligible for that measure in that given calendar year, a requirement that is commonly used in pay-for-performance programs. Performance on the measure was calculated by the registry vendor by calendar year according to technical specifications provided by CMS or the ACR. Since we had 3 years of data, practices could contribute more than 1 observation for each measure analyzed.

The exposure was engagement with the RISE dashboard. Dashboard audit log data were used to identify sessions and actions taken on the dashboard by practice personnel (see “Data sources” described earlier). All dashboard sessions and actions from all individuals within a practice were aggregated into a single practice profile to account for the quality monitoring effort of all users within a practice for each study year. In order to align with the CMS MIPS submission deadline of March 31 and ensure that all sessions and actions on the dashboard pertained to a corresponding performance year, we assessed audit logs for the dashboard from April 1 to March 31 of the following year (Appendix C in Multimedia Appendix 1). For example, for the study year 2020, sessions and actions taken from April 1, 2020, through March 31, 2021, corresponded to the quality measure performance reported for 2020.

We pursued 2 main sets of analyses: “measure-specific” and “global” engagement. The measure-specific analyses were designed to examine the actions (drill-downs, exports) taken by practice personnel for a specific measure; this is the strongest evidence we have that the dashboard was used to facilitate quality improvement activities. First, we identified practice-level drill-down views and drill-down exports for a particular measure. Next, we used the count for these actions, dichotomized at the 90th percentile, to create a binary measure-specific exposure category. In a second analysis, we dichotomized measure-specific actions as any versus none.

The “global engagement” analyses were designed to understand whether regular and sustained engagement with the dashboard across any measure was associated with measure performance.

For the global analyses, we classified practices according to the sessions and actions across all measures based on a published framework for dashboard engagement [20]. Briefly, the BDC framework defines engagement profiles according to the number and consistency of sessions and actions over time; thresholds for minimal, moderate, and most engagement are defined based on median session and action counts for the study sample.

In this study, “session consistency” was defined as the percentage of months with ≥2 sessions (median) during each study year and used as a measure of breadth; “action consistency” was defined as the percentage of months with ≥1 actions (median) during each study year and used as a measure of depth. Actions included drill-down views, drill-down exports, and performance summary exports. Using these 2 metrics, we classified practices into one of 4 global engagement profiles: most, moderate, minimal, and none (Appendix D in Multimedia Appendix 1). Per the BDC framework, “most” engaged practices were defined as those with ≥2 sessions/month for at least 6 months in a study year and ≥1 action/month for at least 6 months during a study year (both were 75th percentiles for the study population). “Moderately” engaged practices were defined as those with some sessions and actions but did not meet the requirements for “most.” “Minimally” engaged practices were defined as those with ≥1 session and 0 action during a study year, and practices with no engagement were those with 0 to 1 session during the study year.

### Covariates

As specified by the BDC framework, we identified several covariates that could affect the relationship between engagement and quality measure performance: EHR vendor, region, total patient count, patient case-mix variables, and study year. We included the EHR vendor as a covariate since there is evidence that EHR can impact the documentation of measure components and affect overall quality measure performance and engagement with the dashboard [22-24]. The total patient count was defined as the number of patients with at least 2 visits in the practice between 2019 and 2022. Patient case-mix variables included the percentage of patients who self-identified as female, median age, the percentage of patients who self-identified as non-Hispanic white, and the percentage of patients with private insurance (the primary payors in the RISE registry). Additional practice-level variables in our analysis included practice type (single-specialty group practice, solo practitioner, and multispecialty group practice) and number of rheumatologists. The number of rheumatologists was derived by linking providers’ National Provider Identifiers to the publicly available “Medicare Physician & Other Practitioners—by Provider Service” file to identify clinicians’ degrees and specialties [25].

### Statistical Analysis

Descriptive statistics were used to summarize the characteristics of the practices and quality measures included in the study. For each measure, we reported the median number of patients in the measure denominator and median performance (%) overall and by measure-specific and global engagement profiles, as well as the mean (SD) number of years a practice was included in the analysis.

We constructed 8 regression models, 1 model for each quality measure, to assess the relationship between dashboard engagement and performance. Although quality measure performance is typically conceptualized as a percentage and can be modeled using linear regression, it is a continuous and bounded response between 0 and 100. This bounded nature violates an assumption of linear regression, which assumes a continuous and unbounded outcome and can lead to unrealistic predictions outside of the allowable range of 0%-100%. To address this, we used binomial generalized linear models for practice-specific counts of patients meeting quality measure criteria, with logit link function and denominators specified by the number of patients, which allowed us to model the proportion of patients who fulfilled the numerator as a bounded response [26].

Additionally, models were adjusted for EHR vendor, study year, and clustering at the practice level to account for repeated measures of the same practice across multiple study years. Odds ratios (ORs) with 95% CI were reported, representing the ratio of quality measure performance for each dashboard engagement profile compared to the referent group, holding all other variables constant.

Two sets of analyses were completed: (1) measure-specific engagement and (2) global engagement (see “Exposures” section). Measure-specific analyses included exposures defined as (1) counts of actions performed on a specific measure, dichotomized at the 90th percentile and (2) collapsing measure-specific actions into a binary category of “any” versus none.

Global analyses included exposures defined as (1) the 4 BDC engagement profiles (most, moderate, minimal, and none); (2) collapsing global engagement profiles into “any” versus none; and (3) using the 4 profiles, we added an interaction term between EHR and the global engagement profiles. We also tested the linear trends between the level of dashboard engagement and performance using linear orthogonal polynomial contrasts.

*P* values <.05 were considered statistically significant. The analytic dataset was created with SAS Enterprise Guide 8.3 (SAS Institute Inc) and analyses were performed using Stata 18 (StataCorp LLC). All figures were created in RStudio (RStudio: Integrated Development for R).

### Ethical Considerations

The Western institutional review board and University of California San Francisco Committee on Human Research (protocol #21-34133) approved this study with waiver of informed consent. The research was of no more than minimal risk to subjects, could not practicably be done without the waiver or alteration, could not practicably be done without identifiable information, and would not adversely affect rights and welfare of subjects with the waiver or alteration. Identifiable data were stored on secure servers and only accessed by institutional review board–approved study staff with appropriate training. As this was a retrospective study, there was no compensation provided to the participants.

## Results

### Practice and Quality Measure Characteristics

A total of 211 RISE practices were included in the study. Over half of the practices (55.5%) were single-specialty practices, 71.1% were in the southern and western regions of the United States, and 43.1% used the EHR vendor NextGen. Practices had a median total patient count of 4132 (IQR 2228‐7811) and were staffed by a median of 2 (IQR 1–4) rheumatologists (Table 1). During their first year included in the study, practices had a median number of sessions of 10 (IQR 4-22). The median number of drill-down views and drill-down exports were 4 (IQR 0‐45) and 0 (IQR 0‐30), respectively. Over half of the practices had “most” or “moderate” levels of global engagement; 20.4% of the practices had “minimal” engagement, and 14.2% had no engagement.

**Figure 1.**
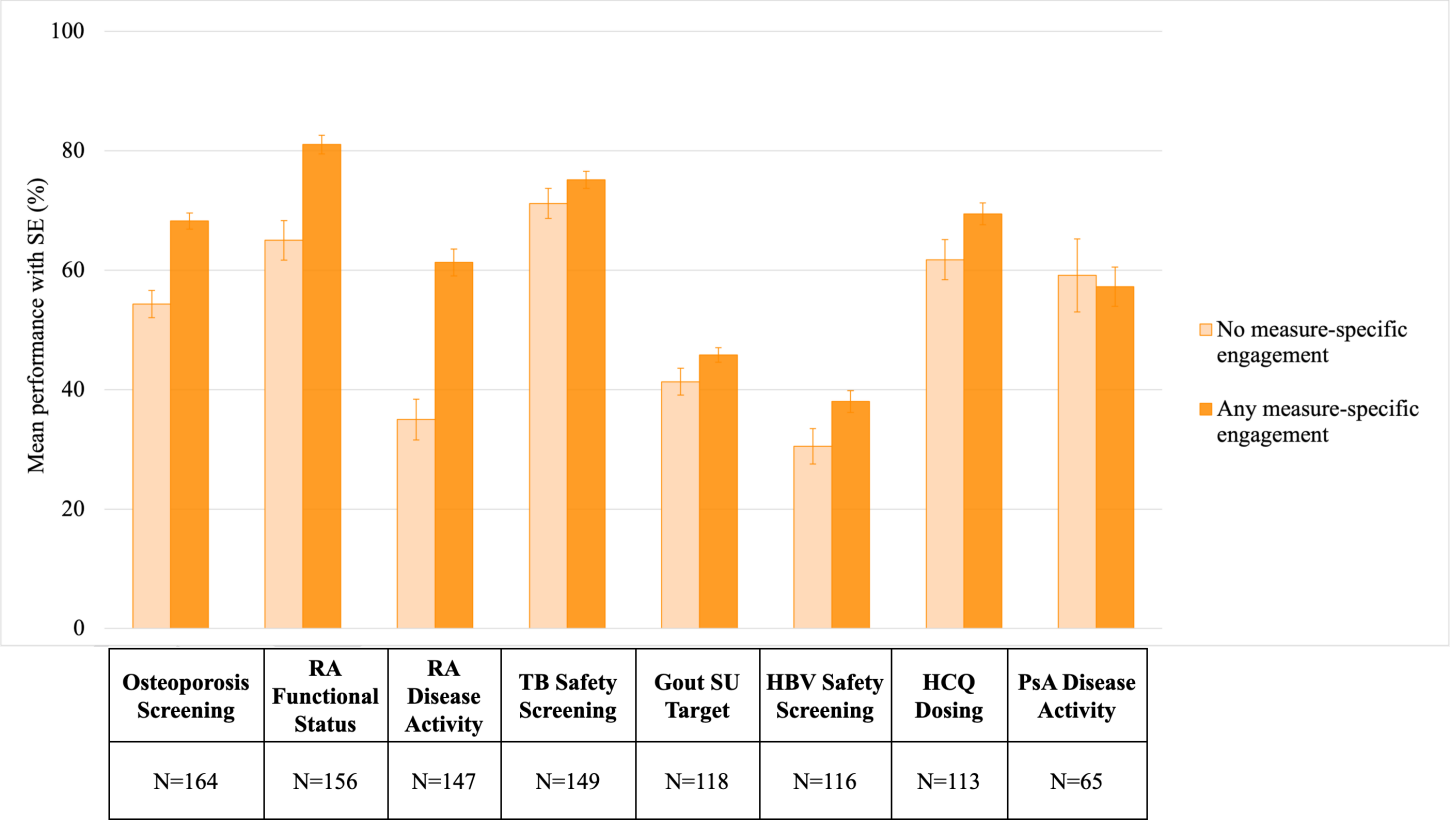
Unadjusted mean (SE) quality measure performance by practice measure-specific engagement during 2020-2022. N indicates the distinct number of practices included in the analysis for each measure; practices could contribute more than 1 observation for each measure analyzed. HBV: hepatitis B virus; HCQ: hydroxychloroquine; PsA: psoriatic arthritis; RA: rheumatoid arthritis; SU: serum urate; TB: tuberculosis.

After requiring practices to have at least 20 patients in the measure denominator for a given measure, 164 out of 211 practices (77.7%) were included in the analysis of osteoporosis screening; analyses of other measures included fewer practices. Additional details regarding the analysis for each measure are shown in Table 2. Overall, we observed substantial variation in quality performance across different measures, ranging from a median performance of 36.8% (IQR 16.3%‐51.5%) for HBV safety screening to 89.3% (IQR 69.1%‐97.4%) for RA functional status assessment (Table 2 and Appendices E1 and E2 in Multimedia Appendix 1). Practices had an average of 2 (SD 1) years of data for each measure. In the unadjusted analysis, we observed higher engagement to be associated with higher performance for most measures in both measure-specific and global engagement analyses (Figures1  2).

**Table 1.** Practice characteristics and dashboard engagement, overall and by global engagement profiles in the first study year.

All included practices		Overall, N=211	Most engaged, n=40	Moderate, n=98	Minimum, n=43	None, n=30
Practice type, n (%)						
	Single-specialty group practice	117 (55.5)	25 (62.5)	56 (57.1)	20 (46.5)	16 (53.3)
	Solo practitioner	71 (33.6)	9 (22.5)	30 (30.6)	21 (48.8)	11 (36.7)
	Multi-specialty group practice	23 (10.9)	6 (15.0)	12 (12.2)	2 (4.7)	3 (10.0)
Number of rheumatologists^a^, median (IQR)		2.0 (1.0‐4.0)	3.0 (1.0‐5.5)	2.0 (1.0‐4.0)	1.0 (1.0‐2.0)	1.5 (1.0‐3.0)
EHR^b^ vendor, n (%)						
	Nextgen	91 (43.1)	14 (35.0)	48 (49.0)	17 (39.5)	12 (40.0)
	eClinicalWorks	31 (14.7)	9 (22.5)	10 (10.2)	7 (16.3)	5 (16.7)
	eMDs	28 (13.3)	7 (17.5)	13 (13.3)	5 (11.6)	3 (10.0)
	Amazing Charts	17 (8.1)	1 (2.5)	8 (8.2)	8 (18.6)	0 (0)
	Other	44 (20.9)	9 (22.5)	19 (19.4)	6 (14.0)	10 (33.3)
Region						
	South	100 (47.4)	18 (45.0)	51 (52.0)	18 (41.9)	13 (43.3)
	West	50 (23.7)	6 (15.0)	22 (22.4)	13 (30.2)	9 (30.0)
	Northeast	31 (14.7)	6 (15.0)	13 (13.3)	8 (18.6)	4 (13.3)
	Midwest	30 (14.2)	10 (25.0)	12 (12.2)	4 (9.3)	4 (13.3)
Practice patient count^c^, median (IQR)		4132 (2228‐7811)	7309 (4336‐10848.5)	4012.5 (2517‐8128)	2502 (1566‐4206)	4503.5 (1723‐7716)
Patient case mix, median (IQR)						
	Percent of female	75.4 (72.6‐77.8)	75.9 (71.8‐77.8)	75.5 (73.0‐77.8)	75.6 (72.1‐77.4)	74.3 (71.8‐78.2)
	Mean age	65.0 (62.0‐67.0)	64.5 (62.0‐66.5)	64.0 (62.0‐67.0)	65.0 (63.0‐69.0)	65.5 (62.0‐68.0)
	Percent non-Hispanic White	72.2 (43.0‐83.9)	76.9 (44.2‐84.8)	70.9 (43.0‐83.8)	75.2 (49.4‐85.1)	61.4 (37.9‐75.8)
	Percent with private insurance	38.9 (30.3‐49.8)	40.2 (35.4‐49.9)	39.1 (30.3‐48.7)	36.8 (26.5‐51.8)	39.1 (31.9‐47.2)
Dashboard engagement in first study year						
	Number of sessions, median (IQR)	10 (4‐22)	33.5 (25.5‐45.5)	7 (11-17)	7 (3-9)	0 (0‐1)
	Number of drill-down views, median (IQR)	4 (0‐45)	161.5 (101.5‐288)	8.5 (0‐26)	0 (0‐0)	0 (0‐0)
	Number of drill-down exports, median (IQR)	0 (0‐30)	34 (0‐236.5)	4.5 (0‐62)	0 (0‐0)	0 (0‐0)
	Number of performance summary exports, median (IQR)	0 (0‐9)	51 (16.5‐151)	0 (0‐8)	0 (0‐0)	0 (0‐0)

a Rheumatologists were derived by linking providers’ National Provider Identifiers (NPIs) to the publicly available “Medicare Physician & Other Practitioners—by Provider Service” file.

b EHR: electronic health record.

c Patients who had at least 2 visits between 2019 and 2022 were included in the patient count.

**Table 2.** Quality measure characteristics.

Measure ID	Measure name	Practices,^a^ N	Measure denominator^b^, median (IQR)	Measure performance^c^ (%), median (IQR)	Measure type	Measure achievement points^d^ (range)	Measure debut in MIPS (year)
QPP39	Screening for osteoporosis for women aged 65‐85 y	164	692.0 (332.0‐1170.0)	65.5 (50.2‐82.4)	Process	3 to 10	2017
QPP178	Rheumatoid arthritis (RA): functional status assessment	156	708.0 (393.0‐1486.0)	89.3 (69.1‐97.4)	Process	3 to 7	2017
QPP177	Rheumatoid arthritis (RA): periodic assessment of disease activity	147	807.0 (415.0‐1642.0)	64.3 (10.3‐83.9)	Process	3 to 10	2017
QPP176	Tuberculosis screening prior to first course of biologic and/or immune response modifier therapy	149	78.0 (38.0‐142.0)	79.6 (62.7‐90.8)	Process	3 to 10	2017
ACR14	Gout: serum urate target	118	72.0 (42.0‐116.0)	46.1 (34.4‐55.4)	Intermediate outcome—high priority	Varies^e^	2020
ACR10	Hepatitis B safety screening	116	199.5 (104.0‐342.0)	36.8 (16.3‐51.5)	Process—high priority	Varies^e^	2020
ACR15	Safe hydroxychloroquine dosing	113	333.0 (168.0‐595.0)	72.6 (58.5‐84.4)	Process—high priority	Varies^e^	2021
ACR12	Disease activity measurement for patients with psoriatic arthritis (PsA)	65	297 (130‐530)	44.8 (9.6‐84.7)	Process	Varies^e^	2020

a Number of unique practices included in measure analysis for at least 1 study year (2020, 2021, or 2022).

b Median (IQR) of patients include ind the measure denominator per practice across all study years (2020‐2022).

c Median (IQR) of percent performance (measure numerator/measure denominator) per practice all study years (2020‐2022).

d Range of possible measure achievement points a practice is eligible to receive based on their performance data submitted to the Merit-based Incentive Payment Program (MIPS). Achievement points contribute to practices’ overall quality performance category score, which impacts their payment adjustment.

e Qualified Clinical Data Registry measures’ maximum eligible achievement points varied by performance period and whether the measure had a benchmark: 2020: practices that met data completeness and submitted ACR14, ACR10, or ACR12 received 3 points per measure, as these were not scored against a benchmark; 2021: practices that met data completeness and submitted ACR14, ACR10, ACR12, or ACR15 received 3 points per measures, as these were not scored against a benchmark; 2022: practices that met completeness and submitted ACR15 received 5 points for the measure because ACR15 was in its second year in MIPS and lacking a reliable benchmark. Practices that met data completeness and submitted ACR14, ACR10, or ACR12 received between 3 and 10 points based on their performance since these measures were now scored against a benchmark.

**Figure 2.**
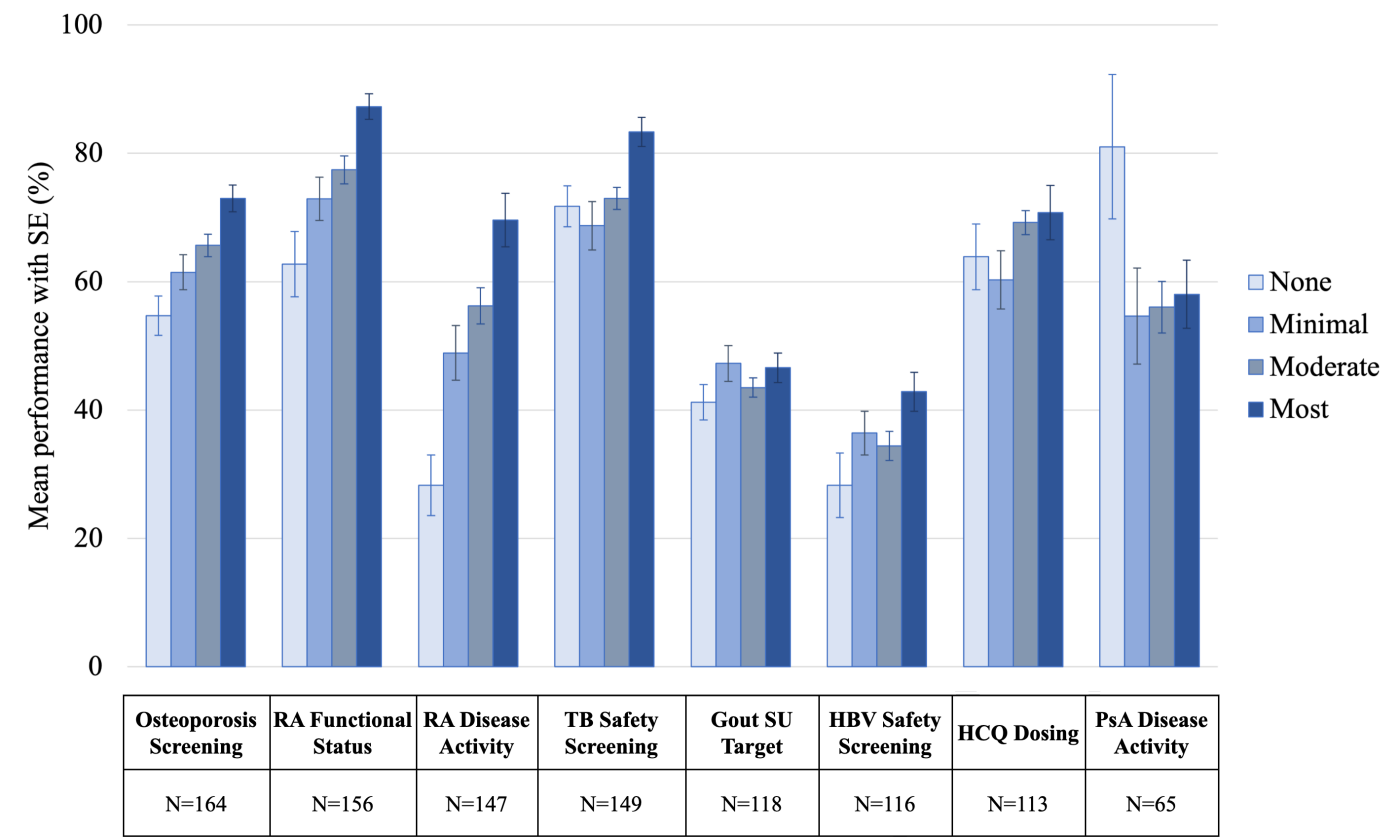
Unadjusted mean (SE) quality measure performance by practice global engagement profile during 2020-2022. N indicates the distinct number of practices included in the analysis for each measure; practices could contribute more than 1 observation for each measure analyzed. HBV: hepatitis B virus; HCQ: hydroxychloroquine; PsA: psoriatic arthritis; RA: rheumatoid arthritis; SU: serum urate; TB: tuberculosis.

### Measure-Specific Engagement Analyses

In the model that included counts of actions performed on a specific measure, dichotomized at the 90th percentile, we observed a positive association of both drill-down views and drill-down exports with performance on 7 of the 8 measures, although this was only statistically significant for QPP 177 (RA: periodic assessment of disease activity; see Appendix F in Multimedia Appendix 1). In the measure-specific any versus none analysis, we observed a similar pattern (RA periodic disease activity assessment (OR 1.93, 95% CI 1.12‐3.34; Table 3).

**Table 3.** Measure-specific analysis, binomial generalized linear models: examining the association between measure-specific engagement and quality performance^a^.

Any measure-specific dashboard engagement?	MIPS^b^ QPP^c^ measures								QCDR^d^ measures (ACR^e^)							
	QPP39: osteoporosis screening (164 practices)		QPP178: RA^f^ functional status assessment (156 practices)		QPP177: RA periodic assessment of disease activity (147)		QPP176: TB^g^ safety screening (149 practices)		ACCR14: gout serum urate target (118 practices)		ACR10: HBV^h^ safety screening		ACR15: safe HCQ^i^ dosing		ACR12: PsA^j^ disease activity measurement (65 practices)	
	Unadjusted OR^k^ (95% CI)	Adjusted OR (95% CI)	Unadjusted OR (95% CI)	Adjusted OR (95% CI)	Unadjusted OR (95% CI)	Adjusted OR (95% CI)	Unadjusted OR (95% CI)	Adjusted OR (95% CI)	Unadjusted OR (95% CI)	Adjusted OR (95% CI)	Unadjusted OR (95% CI)	Adjusted OR (95% CI)	Unadjusted OR (95% CI)	Adjusted OR (95% CI)	Unadjusted OR (95% CI)	Adjusted OR (95% CI)
No	Reference	Reference	Reference	Reference	Reference	Reference	Reference	Reference	Reference	Reference	Reference	Reference	Reference	Reference	Reference	Reference
Yes	1.23 (0.95‐1.58)	1.26 (0.99‐1.61)	1.50 (0.90‐2.50)	1.34 (0.80‐2.25)	1.91^l^ (1.11‐3.30)	1.93^l^ (1.12‐3.34)	1.00 (0.63‐1.59)	1.05 (0.68‐1.63)	1.01 (0.80‐1.26)	1.08 (0.88‐1.31)	1.09 (0.78‐1.54)	1.01 (0.71‐1.43)	0.85 (0.58‐1.24)	0.81 (0.57‐1.14)	1.13 (0.57‐2.23)	1.19 (0.61‐2.32)

a All models were binomial generalized linear models for practice-specific counts of patients meeting quality criteria (quality performance), with logit link function and denominators specified by the number of patients and adjusted for electronic health record vendor, year, and clustering by practice; measure-specific engagement was determined by practices’ dashboard engagement on a particular measure.

b MIPS: Merit-based Incentive Payment System.

c QPP: Quality Payment Program.

d QCDR: Qualified Clinical Data Registry.

e ACR: American College of Rheumatology.

f RA: rheumatoid arthritis.

g TB: tuberculosis.

h HBV: hepatitis B virus.

i HCQ: hydroxychloroquine.

j PsA: psoriatic arthritis.

k OR: odds ratio.

l *P*<.05.

### Global Engagement Analyses

**Figure 3.**
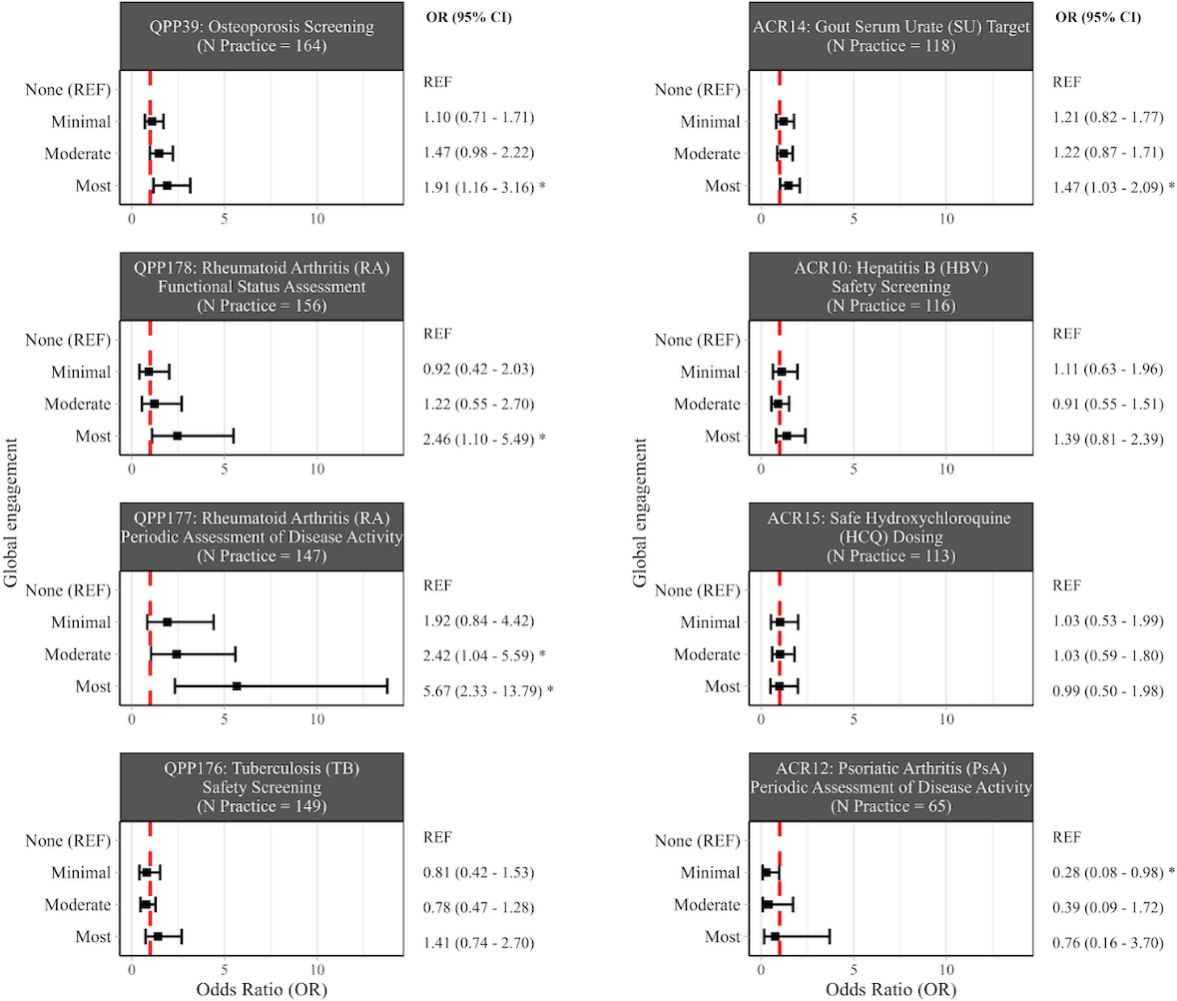
Adjusted odds ratios (ORs) for the relationship between Rheumatology Informatics System for Effectiveness (RISE) dashboard global engagement and performance on quality measures. This figure displays the adjusted ORs and 95% CIs from binomial generalized linear models for practice-specific counts of patients meeting measure criteria, with logit link function and denominators specified by the number of patients. All models were also adjusted for electronic health record and year. ORs are denoted by the black square, and the upper and lower bounds of the CI are denoted by the black vertical lines. *P<.05.

Figure 3 shows the results of the 8 binomial generalized linear regression analyses for the global engagement analyses. ORs with 95% CI are shown for each level of engagement using “None” as the reference category. Even after adjustment for covariates, practices with more engagement had significantly higher performance compared to practices with no dashboard engagement for 4 of the 8 measures examined (osteoporosis screening, RA functional status assessment, RA periodic assessment of disease activity, and gout SU target; model output details in Appendix G in Multimedia Appendix 1). We also detected a significant “dose-response” relationship between engagement and performance for these measures, as indicated by the significant linear polynomial contrast (*P*=.004, .02,<.001, and .04, respectively, for trend). ORs were not statistically different across engagement categories for the HBV safety screening, safe HCQ dosing, TB safety screening, and PsA periodic assessment of disease activity measures.

In the analysis that collapsed all global engagement categories into “any” versus none, practices with any engagement had higher performance on 6 of the 8 measures, although only 1 was statistically significant (RA periodic disease activity assessment (OR 2.91, 95% CI 1.29‐6.61; Appendix H in Multimedia Appendix 1). When we repeated the primary global engagement analysis with an interaction term for EHR and global engagement profile, we observed a significant interaction for 5 measures (osteoporosis screening, RA functional status assessment, RA periodic disease activity assessment, gout SU target, and safe HCQ dosing, *P*-interaction=.009, .03, <.001,<.001, and <.001, respectively).

## Discussion

Understanding the ways in which practices engage with QCDR dashboards and whether engagement is associated with quality measure performance is critical for determining whether the significant investments in dashboard development and maintenance are justified. In this study, we used audit log data and applied the BDC framework to create profiles of dashboard engagement by practices participating in the RISE registry. We found a cross-sectional relationship between higher levels of engagement with the QCDR dashboard and higher performance on some, but not all, of the assessed quality measures, with the strongest associations for osteoporosis screening, RA functional status assessment, and RA periodic disease activity assessment. The cross-sectional design of this study limits the ability to establish causality, and it is possible that dashboard engagement could be both a driver and a result of better performance. However, these findings suggest additional work is warranted to understand how the dashboard may facilitate performance improvement or whether it is merely a marker for high-performing practices.

To our knowledge, this is the first study to examine engagement with a QCDR dashboard and its association with quality measure performance. While some studies have reported that clinicians participating in QCDRs have shown improved performance over time and have higher MIPS quality performance scores than those who report separately as individuals or groups, it is unclear whether this higher performance is due to the additional support from QCDR dashboards themselves or the financial incentives tied more broadly to specialty-specific quality reporting through QCDRs [4,5,27-31]. Studies evaluating the effects of dashboards on quality of care typically compare performance metrics before and after the dashboard’s introduction and do not consistently report clinician engagement [7-15]. Our study advances previous research by capturing the multidimensional nature of dashboard engagement and using standardized quality performance measures that make our findings more generalizable to future studies in this emerging field.

The relationship between dashboard engagement and performance was not uniform across the quality measures we examined. For most of the measures, we did observe a general pattern of higher performance associated with more engagement and a potential “dose response” for some, but this relationship was strongest (both statistically significant and robust to adjustment for covariates) for QPP177: RA: periodic assessment of disease activity. This observation raises the question of whether something is different about this measure compared to the others: First, this measure included more patients in its denominator than many of the others and more practices contributing data, which likely improved the precision of the OR estimate. Second, as seen in Table 2, these measures had the potential to earn more MIPS points compared to other measures, which may have motivated clinicians to be more attentive to these measures [13,32,33]. Third, this measure may be particularly amenable to quality improvement efforts, since it requires that RA disease activity be assessed at 50% of visits during the measurement year; therefore, if patients have missed documentation of RA disease activity at prior visits, it is possible to improve the score by documenting it at upcoming visits.

Leveraging QCDR audit log data is novel and offers a robust way to objectively quantify patterns of dashboard use. Practices’ RISE dashboard use varied widely, and audit log data enabled us to capture the frequency and consistency of dashboard utilization to the level of the specific measure. Nevertheless, our classification of engagement may not have fully captured the quantity or quality of interactions with the RISE dashboard; for example, we did not have information on session duration or the time spent interacting with particular measures within the dashboard. It is also unknown what specific interventions are taken by clinicians as a result of using the dashboard, although patient list views and downloads imply (but do not prove) that this information is used to review patient charts and actions to fulfill numerator requirements. Audit log data is not suited to capturing differences in practice workflows and culture or external sources of data that could be used for quality improvement. While we did adjust for EHR vendor in the multivariate model, different implementations of the same EHR can have different native tools for quality monitoring that could potentially replace the tools available through the RISE dashboard [34-36]. In addition, there may be other unmeasured factors that could influence both dashboard engagement and quality measure performance. Finally, our relatively strict inclusion criteria for this analysis may limit generalizability to all rheumatology practices, other specialties, or QCDRs with different designs or functionalities.

In summary, using user audit logs, we classified practices according to level of engagement with a web-based QCDR dashboard and examined the association between engagement and performance on a series of specialty-specific quality measures. Although our findings suggest that, in general, higher levels of engagement with the QCDR dashboard are associated with higher quality measure performance, it remains unclear whether the dashboard drives quality improvement or simply serves as a marker for practices that are already high-performing. Future research using more robust methods such as cluster randomized trials should explore the potential mechanisms underlying this association, including whether specific dashboard features, such as patient-level reports or performance benchmarking, play a direct role in enhancing quality outcomes. If the dashboard does contribute to quality improvement for certain measures, efforts should focus on optimizing its design and functionality to extend these benefits across a broader range of quality measures. Tailored interventions such as staff training or automation of alerts could enhance the utility of a QCDR dashboard and ensure that it serves as a cost-effective mechanism for sustained quality improvement.

## Supplementary material

10.2196/72709 Multimedia Appendix 1 Additional figures and tables.

## Abbreviations

ACR: American College of Rheumatology
BDC: breadth-depth-context
CMS: Centers for Medicare and Medicaid Services
EHR: electronic health record
HBV: hepatitis B virus
HCQ: hydroxychloroquine
MIPS: Merit-based Incentive Payment System
OR: odds ratio
PsA: psoriatic arthritis
QCDR: Qualified Clinical Data Registry
QPP: Quality Payment Program
RA: rheumatoid arthritis
RISE: Rheumatology Informatics System for Effectiveness
SU: serum urate
TB: tuberculosis
