# Supplementary material for: Association of Qualified Clinical Data Registry Clinician Dashboard Engagement With Performance on Quality-of-Care Measures: Cross-Sectional Analysis
Source: J Med Internet Res. 2025 Sep 4;27:e72709. doi: 10.2196/72709 (PMC13006825; doi:10.2196/72709)
Supplement: Multimedia Appendix 1 [file jmir-v27-e72709-s001.docx]

**APPENDIX A. Overview of RISE registry clinician dashboard**

**
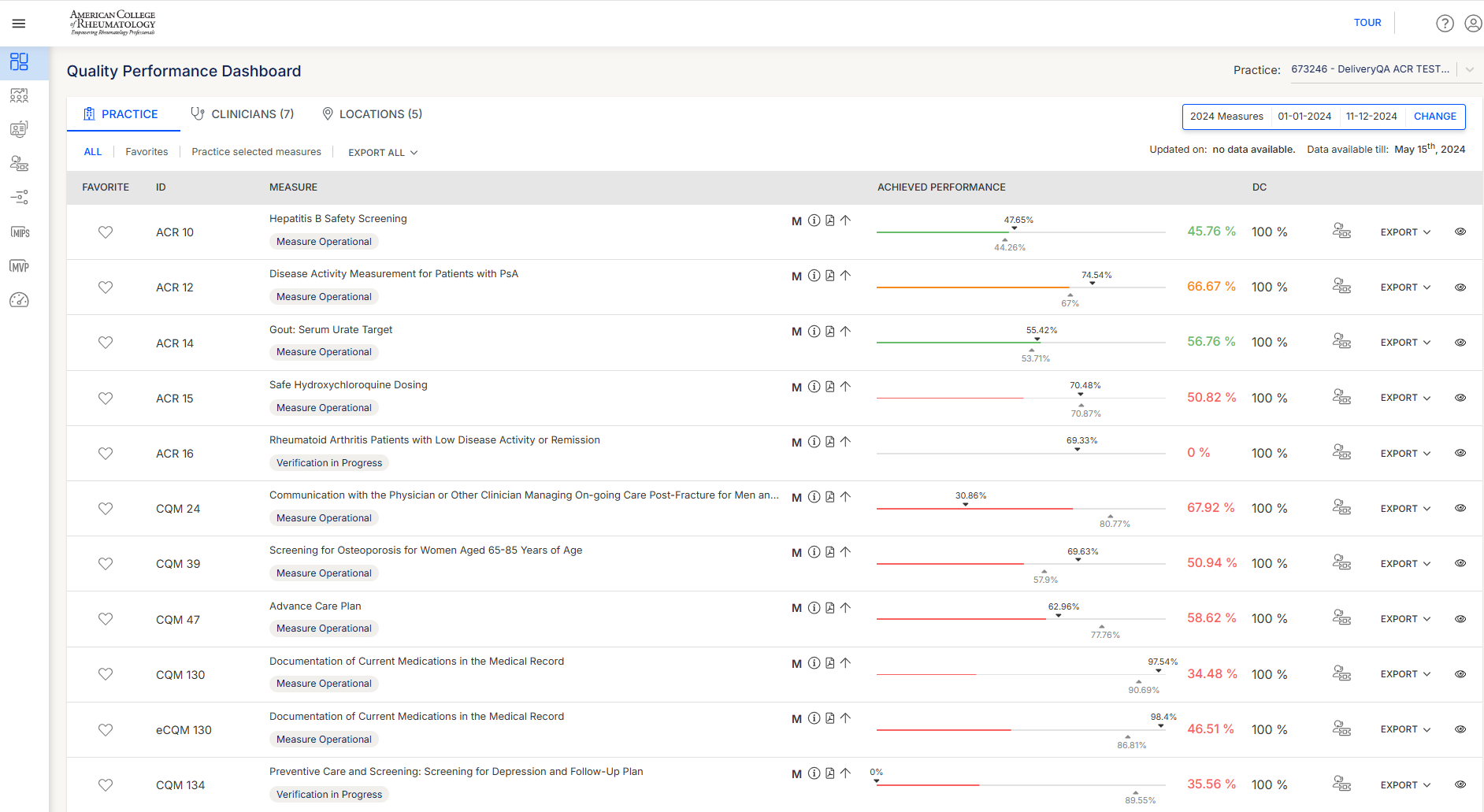
**

| **RISE clinician dashboard features** | | **Description** | **Application in quality improvement** |
| --- | --- | --- | --- |
| **Dashboard session** | | A user login to the dashboard. Upon logging in, users will be brought to the landing page. |  |
|  | Landing page | The dashboard landing page displays aggregated performance on each of the quality measures monitored by the practice, along with the RISE registry average performance as benchmark comparison. | Offers an overview of the practice’s performance relative to the registry benchmarks, helping identify gaps and prioritize areas needed improvement. |
| **Dashboard action** | | Users can complete additional advanced dashboard functions (actions) from the initial landing page. |  |
|  | Drill-down view | View patient-level data on any number of user selected quality measures. A drilldown view is required prior to a drilldown export action. | Enables users to identify specific patients contributing to performance gaps and to target interventions more precisely. |
|  | Drill-down export | Download patient-level data on any number of user selected quality measures | Allows offline review, care planning, and integration with other practice tools for quality improvement. |
|  | Performance summary report | Generate a summary-level report on overall performance for any number of user selected quality measures | Supports high-level progress tracking and communication with leadership for internal QI initiatives. |

**APPENDIX B. Measure specifications**

| **Measure ID** | **Measure name** | **Measure specification** |
| --- | --- | --- |
| QPP39 | Screening for Osteoporosis for Women Aged 65-85 Years of Age | Percentage of female patients aged 65-85 years of age who ever had a central dual-energy X-ray absorptiometry (DXA) to check for osteoporosis. |
| QPP178 | Rheumatoid Arthritis (RA): Functional Status Assessment | Percentage of RA patients with at least one functional status assessment during measurement year. |
| QPP177 | Rheumatoid Arthritis (RA): Periodic Assessment of Disease Activity | Percentage of RA patients with disease activity assessments at ≥ 50% of visits during the measurement year. |
| QPP176 | Tuberculosis Screening Prior to First Course of Biologic and/or Immune Response Modifier Therapy | Percentage of new biologic DMARD users with a TB test completed in the preceding 12-month period. |
| ACR14 | Gout: Serum Urate Target | Percentage of patients with gout on urate lowering therapy for at least 12 months with most recent serum urate at target of < 6 mg/dL. |
| ACR10 | Hepatitis B Safety Screening | Percentage of patients initiating a new conventional or biologic DMARD with HBV screening (HBV surface antigen) in the preceding 12-month period. |
| ACR15 | Safe Hydroxychloroquine (HCQ) Dosing | Percentage of patients receiving HCQ with an average daily dose at ≤ 5 mg/kg/day. |
| ACR12 | Disease Activity Measurement for Patients with Psoratic Arthritis (PsA) | Percentage of PsA patients with disease activity assessments at ≥ 50% of visits during the measurement year. |

**APPENDIX C. Distribution of dashboard sessions align with MIPS submission deadlines**

**
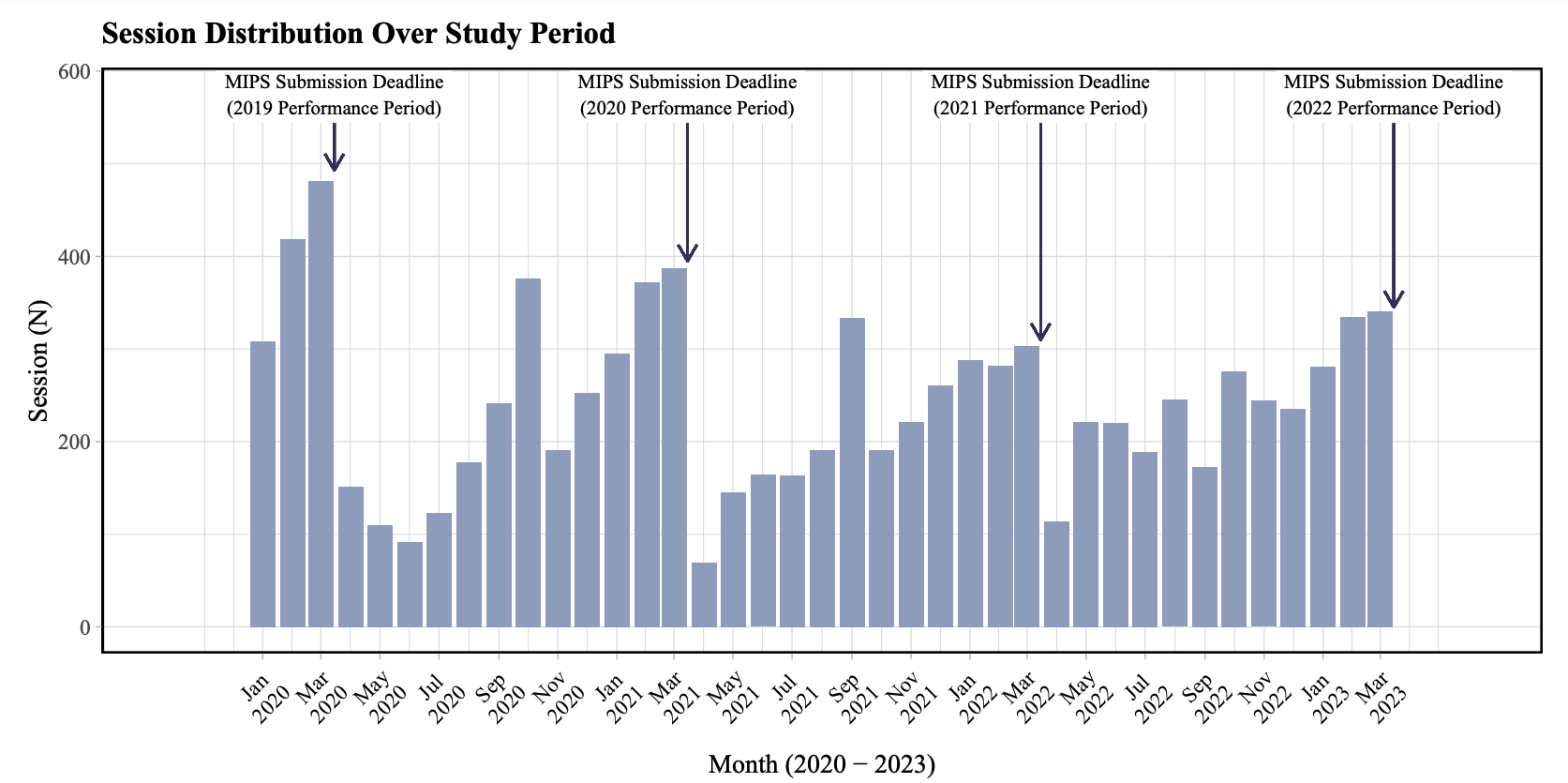
**

This figure showed the distribution of dashboard sessions over the course of the study period (2020 – 2023). In general, sessions seemed to increase up until the MIPS submission deadline of March 31^st^.

**APPENDIX D. Employing the Breadth-Depth-Context (BDC) framework to classify profiles of RISE dashboard engagement**

This figure displayed a brief overview of the key components of the Breadth-Depth-Context framework used to classify profiles of RISE dashboard engagement. Based on our experience with quality improvement programs, we believed that the most successful programs monitor performance regularly and use patient-level data to identify gaps in care. Therefore, we decided to use the metric of session consistency to assess the breadth dimension of dashboard use and measure-action consistency to assess the depth dimension of dashboard use. We determined the cut-offs for high breadth and depth empirically via conventional statistics to identify the median of sessions and measure actions per month among practices with at least 1 session: 2 sessions and 1 measure-action, respectively. As such, we classified most engaged users as those with ≥2 sessions/month for at least 6 months (high breadth), and ≥1 measure-action/month for at least 6 months (high depth) during a study year. We classified moderate engaged users as those with some sessions and measure-actions but did not meet the requirements of the most engaged users (high breadth and low depth, low breadth and high depth, or low breadth and low depth) during a study year. Minimally engaged users were classified as those with ≥1 session and 0 measure-actions (some breadth and no depth) during a study year, and users with no engagement were those with 0 to 1 session during the study year (no breadth and no depth).

**Appendix E1. Quality measure performance (%) overall, and stratified by global dashboard engagement during 2020-2022^a^**

| **MIPS Clinical Quality Measures (QPP)** | | **QPP39: Osteoporosis Screening** | | **QPP178: RA Functional  Status Assessment** | | **QPP177: RA Periodic Assessment of Disease Activity** | | **QPP176: TB Safety Screening** | |  |
| --- | --- | --- | --- | --- | --- | --- | --- | --- | --- | --- |
|  |  |  |  |  |  |  |  |  |  |  |
|  |  |  |  |  |  |  |  |  |  |  |
| Number of years included, mean (SD) | | 2 (1) | | 2 (1) | | 2 (1) | | 2 (1) | |  |
| **Overall performance, median (IQR)** | | 65.5 (50.2 - 82.4) | | 89.3 (69.1 - 97.4) | | 64.3 (10.3 - 83.9) | | 79.6 (62.7 - 90.8) | |  |
| **Global dashboard engagement** | | n | performance, median (IQR) | n | performance, median (IQR) | n | performance, median (IQR) | n | performance, median (IQR) |  |
|  | None | 80 | 58.1 (38.0 - 77.6) | 58 | 75.2 (24.7 - 99.0) | 56 | 1.1 (0.0 - 61.8) | 50 | 77.2 (65.5 - 86.6) |  |
|  | Minimal | 100 | 65.6 (43.0 - 82.1) | 94 | 88.9 (57.6 - 97.4) | 80 | 61.6 (3.6 - 81.6) | 53 | 79.4 (45.5 - 93.5) |  |
|  | Moderate | 167 | 66.3 (53.2 - 82.9) | 168 | 89.3 (72.8 - 96.7) | 140 | 64.6 (24.0 - 82.9) | 146 | 76.1 (59.3 - 89.6) |  |
|  | Most | 60 | 73.1 (60.1 - 87.0) | 61 | 91.8 (82.6 - 96.8) | 57 | 83.6 (66.4 - 91.4) | 57 | 87.7 (77.8 - 95.7) |  |
| **QCDR Measures (ACR)** | | **ACR14: Gout SU Target** | | **ACR10: HBV Safety Screening** | | **ACR15: Safe HCQ Dosing** | | **ACR12: PsA Disease  Activity Measurement** | |  |
|  |  |  |  |  |  |  |  |  |  |  |
|  |  |  |  |  |  |  |  |  |  |  |
| Number of years included, mean (SD) | | 2 (1) | | 2 (1) | | 2 (1) | | 2 (1) | |  |
| **Overall performance, median (IQR)** | | 46.1 (34.4 - 55.4) | | 36.8 (16.3 - 51.5) | | 72.6 (58.5 - 84.4) | | 65.1 (24.5 - 89.7) | |  |
| **Global dashboard engagement** | | n | performance, median (IQR) | n | performance, median (IQR) | n | performance, median (IQR) | n | performance, median (IQR) |  |
|  | None | 29 | 40.3 (30.3 - 52.7) | 20 | 25.9 (7.5 - 48.5) | 23 | 65.1 (53.0 - 80.8) | 8 | 96.1 (75.9 - 98.6) |  |
|  | Minimal | 50 | 47.9 (36.4 - 62.5) | 48 | 36.4 (17.4 - 53.1) | 50 | 68.7 (46.3 - 83.6) | 24 | 61.3 (13.1 - 89.0) |  |
|  | Moderate | 116 | 45.2 (33.3 - 54.2) | 105 | 34.6 (13.8 - 50.5) | 118 | 72.0 (60.4 - 83.8) | 76 | 61.4 (20.3 - 90.4) |  |
|  | Most | 46 | 49.0 (36.9 - 57.3) | 37 | 42.2 (30.8 - 55.5) | 44 | 80.2 (70.1 - 86.9) | 35 | 65.3 (38.1 - 87.3) |  |

**Appendix E2. Quality measure performance (%) overall, and stratified by measure-specific dashboard engagement during 2020-2022^a^**

| **MIPS Clinical Quality Measures (QPP)** | | **QPP39: Osteoporosis Screening** | | **QPP178: RA Functional  Status Assessment** | | **QPP177: RA Periodic Assessment of Disease Activity** | | **QPP176: TB Safety Screening** | |  |
| --- | --- | --- | --- | --- | --- | --- | --- | --- | --- | --- |
|  |  |  |  |  |  |  |  |  |  |  |
|  |  |  |  |  |  |  |  |  |  |  |
| Number of years included, mean (SD) | | 2 (1) | | 2 (1) | | 2 (1) | | 2 (1) | |  |
| **Overall performance, median (IQR)** | | 65.5 (50.2 - 82.4) | | 89.3 (69.1 - 97.4) | | 64.3 (10.3 - 83.9) | | 79.6 (62.7 - 90.8) | |  |
| **Any measure-specific dashboard engagement** | | n | performance, median (IQR) | n | performance, median (IQR) | n | performance, median (IQR) | n | performance, median (IQR) |  |
|  | No | 137 | 57.6 (37.1 - 77.4) | 128 | 79.2 (27.5 - 98.5) | 117 | 19.7 (0.0 - 73.9) | 90 | 77.2 (56.9 - 88.5) |  |
|  | Yes | 270 | 70.2 (55.2 - 86.0) | 253 | 90.6 (76.5 - 96.8) | 216 | 71.7 (29.3 - 87.7) | 216 | 80.5 (63.8 - 92.5) |  |
| **QCDR Measures (ACR)** |  | **ACR14: Gout SU Target** | | **ACR10: HBV Safety Screening** | | **ACR15: Safe HCQ Dosing** | | **ACR12: PsA Disease  Activity Measurement** | |  |
|  |  |  |  |  |  |  |  |  |  |  |
|  |  |  |  |  |  |  |  |  |  |  |
| Number of years included, mean (SD) | | 2 (1) | | 2 (1) | | 2 (1) | | 2 (1) | |  |
| **Overall performance, median (IQR)** | | 46.1 (34.4 - 55.4) | | 36.8 (16.3 - 51.5) | | 72.6 (58.5 - 84.4) | | 65.1 (24.5 - 89.7) | |  |
| **Any measure-specific dashboard engagement** | | n | performance, median (IQR) | n | performance, median (IQR) | n | performance, median (IQR) | n | performance, median (IQR) |  |
|  | No | 65 | 40.3 (33.3 - 53.6) | 63 | 28.2 (10.1 - 48.9) | 72 | 67.1 (47.5 - 84.7) | 34 | 69.0 (28.8 - 93.4) |  |
|  | Yes | 176 | 46.6 (35.4 - 56.6) | 147 | 38.1 (21.1 - 53.5) | 163 | 74.0 (61.8 - 84.4) | 109 | 63.6 (24.5 - 89.0) |  |

**^a^**These tables displayed the median (IQR) of performance (%) across the study period per rheumatology-specific quality measure, overall and stratified by global and measure-specific dashboard engagement. The numbers of practices in each engagement for each measure (n) were not mutually exclusive, because engagement could change in different study years.

**APPENDIX F. Measure-specific engagement analysis, binomial generalized linear models: examining the association between specific actions and quality performance^a^**

| **MIPS clinical quality measures (QPP)** | | **QPP39: Osteoporosis screening  (N practice = 164)** | **QPP178: RA functional status assessment (N practice = 156)** | **QPP177: RA periodic assessment of disease activity (N practice = 147)** | **QPP176: TB safety screening  (N practice = 149)** |
| --- | --- | --- | --- | --- | --- |
| **Characteristics** |  | Adjusted | Adjusted | Adjusted | Adjusted |
|  |  | ORs (95% CI) | ORs (95% CI) | ORs (95% CI) | ORs (95% CI) |
| Number of drill-down views ≥ 90th percentile, yes | | 1.27 (0.84 - 1.91) | 1.68 (0.91 - 3.11) | 2.31 (1.24 - 4.29)* | 1.14 (0.72 - 1.79) |
| Number of drill-down exports ≥ 90th percentile, yes | | 1.09 (0.82 - 1.45) | 1.24 (0.73 - 2.12) | 0.93 (0.40 - 2.13) | 1.40 (0.58 - 3.38) |
| **EHR vendor** |  |  |  |  |  |
|  | Nextgen | REF | REF | REF | REF |
|  | Amazing Charts | 0.89 (0.44 - 1.80) | 0.30 (0.07 - 1.24) | 1.48 (0.80 - 2.75) | 0.43 (0.20 - 0.92) |
|  | eClinicalWorks | 1.61 (1.05 - 2.49)* | 1.12 (0.57 - 2.19) | 0.90 (0.45 - 1.81) | 1.14 (0.45 - 2.89) |
|  | eMDS | 1.40 (0.82 - 2.38) | 0.62 (0.30 - 1.29) | 0.80 (0.31 - 2.06) | 1.83 (0.67 - 4.96) |
|  | other | 0.74 (0.48 - 1.16) | 0.76 (0.36 - 1.61) | 0.49 (0.21 - 1.16) | 1.31 (0.56 - 3.10) |
| **Year** |  |  |  |  |  |
|  | 2020 | REF | REF | REF | REF |
|  | 2021 | 1.02 (0.88 - 1.19) | 0.78 (0.59 - 1.01) | 1.00 (0.76 - 1.32) | 0.97 (0.68 - 1.39) |
|  | 2022 | 1.40 (1.18 - 1.66)* | 1.19 (0.88 - 1.61) | 1.29 (0.96 - 1.73) | 1.87 (1.27 - 2.75)* |
| **QCDR measures (ACR)** | | **ACR14: Gout SU target  (N practice = 118)** | **ACR10: HBV safety screening  (N practice = 116)** | **ACR15: Safe HCQ dosing  (N practice = 113)** | **ACR12: PsA disease activity measurement (N practice = 65)** |
| **Characteristics** |  | Adjusted | Adjusted | Adjusted | Adjusted |
|  |  | ORs (95% CI) | ORs (95% CI) | ORs (95% CI) | ORs (95% CI) |
| Number of drill-down views >= 90th percentile, yes | | 1.11 (0.82 - 1.50) | 1.22 (0.76 - 1.94) | 1.58 (0.92 - 2.71) | 1.35 (0.55 - 3.31) |
| Number of drill-down exports >= 90th percentile, yes | | 1.02 (0.78 - 1.33) | 1.10 (0.76 - 1.59) | 1.25 (0.63 - 2.50) | 0.67 (0.23 - 1.97) |
| **EHR vendor** |  |  |  |  |  |
|  | Nextgen | REF | REF | REF | REF |
|  | Amazing Charts | 1.22 (0.75 - 2.00) | 0.91 (0.55 - 1.51) | 0.51 (0.31 - 0.82) | 0.56 (0.22 - 1.38) |
|  | eClinicalWorks | 1.62 (1.17 - 2.24)* | 0.54 (0.34 - 0.84) | 0.46 (0.21 - 1.00) | 1.62 (0.53 - 4.95) |
|  | eMDS | 0.87 (0.61 - 1.25) | 0.87 (0.36 - 2.12) | 1.36 (1.05 - 1.75)* | 1.81 (0.75 - 4.35) |
|  | other | 1.06 (0.80 - 1.42) | 0.48 (0.28 - 0.81) | 1.17 (0.68 - 2.02) | 0.67 (0.33 - 1.36) |
| **Year** |  |  |  |  |  |
|  | 2020 | REF | REF | REF | REF |
|  | 2021 | 1.05 (0.91 - 1.23) | 0.67 (0.47 - 0.95) | 0.66 (0.39 - 1.11) | 2.03 (1.29 - 3.18)* |
|  | 2022 | 1.26 (1.04 - 1.54)* | 1.50 (1.03 - 2.18)* | 0.74 (0.42 - 1.30) | 2.54 (1.41 - 4.58)* |

^a^All models were binomial generalized linear models for practice-specific counts of patients meeting quality criteria (quality performance), with logit link function and denominators specified by the number of patients and adjusted for EHR vendor, year, and clustering by practice; measure-specific engagement was determined by practices' dashboard engagement with a particular measure.

**P* <.05.

**APPENDIX G. Global engagement analysis, binomial generalized linear models: examining the association between global engagement and quality performance^a^**

| **MIPS clinical quality measures (QPP)** | | **QPP39: Osteoporosis screening  (N practice = 164)** | | **QPP178: RA functional status assessment (N practice = 156)** | | **QPP177: RA periodic assessment of disease activity (N practice = 147)** | | **QPP176: TB safety screening  (N practice = 149)** | |  |
| --- | --- | --- | --- | --- | --- | --- | --- | --- | --- | --- |
|  |  |  |  |  |  |  |  |  |  |  |
|  |  |  |  |  |  |  |  |  |  |  |
| **Characteristics** | | Unadjusted | Adjusted | Unadjusted | Adjusted | Unadjusted | Adjusted | Unadjusted | Adjusted |  |
|  |  | ORs (95% CI) | ORs (95% CI) | ORs (95% CI) | ORs (95% CI) | ORs (95% CI) | ORs (95% CI) | ORs (95% CI) | ORs (95% CI) |  |
| **Global dashboard engagement** | | | | | | | | | |  |
|  | None | REF | REF | REF | REF | REF | REF | REF | REF |  |
|  | Minimal | 1.32 (0.81 - 2.13) | 1.10 (0.71 - 1.71) | 0.80 (0.34 - 1.86) | 0.92 (0.42 - 2.03) | 2.12 (0.89 - 5.04) | 1.92 (0.84 - 4.42) | 0.86 (0.49 - 1.50) | 0.81 (0.42 - 1.53) |  |
|  | Moderate | 1.58 (1.03 - 2.43)* | 1.47 (0.98 - 2.22) | 1.23 (0.54 - 2.77) | 1.22 (0.55 - 2.70) | 2.60 (1.07 - 6.29)* | 2.42 (1.04 - 5.59)* | 0.86 (0.52 - 1.42) | 0.78 (0.47 - 1.28) |  |
|  | Most | 2.06 (1.23 - 3.45)* | 1.91 (1.16 - 3.16)* | 2.45 (1.07 - 5.60)* | 2.46 (1.10 - 5.49)* | 5.84 (2.32 - 14.71)* | 5.67 (2.33 - 13.79)* | 1.51 (0.71 - 3.19) | 1.41 (0.74 - 2.70) |  |
| **EHR vendor** | | | | | | | | | |  |
|  | Nextgen | REF | REF | REF | REF | REF | REF | REF | REF |  |
|  | Amazing Charts | 0.84 (0.41 - 1.70) | 0.99 (0.49 - 1.98) | 0.27 (0.06 - 1.17) | 0.33 (0.09 - 1.31) | 1.59 (0.81 - 3.12) | 1.94 (0.99 - 3.80) | 0.40 (0.16 - 0.99)* | 0.48 (0.19 - 1.17) |  |
|  | eClinicalWorks | 1.56 (1.02 - 2.40)* | 1.67 (1.10 - 2.54)* | 1.12 (0.56 - 2.27) | 1.02 (0.54 - 1.92) | 1.03 (0.49 - 2.15) | 0.96 (0.47 - 1.93) | 1.19 (0.41 - 3.52) | 1.07 (0.39 - 2.98) |  |
|  | eMDS | 1.39 (0.82 - 2.36) | 1.37 (0.85 - 2.22) | 0.61 (0.30 - 1.25) | 0.59 (0.29 - 1.19) | 0.90 (0.35 - 2.27) | 0.80 (0.32 - 1.97) | 2.17 (0.68 - 6.91) | 1.95 (0.65 - 5.93) |  |
|  | other | 0.75 (0.48 - 1.17) | 0.80 (0.52 - 1.21) | 0.78 (0.37 - 1.66) | 0.82 (0.40 - 1.67) | 0.63 (0.25 - 1.56) | 0.75 (0.36 - 1.58) | 1.26 (0.54 - 2.92) | 1.19 (0.57 - 2.44) |  |
| **Year** | | | | | | | | | |  |
|  | 2020 | REF | REF | REF | REF | REF | REF | REF | REF |  |
|  | 2021 | 0.98 (0.86 - 1.11) | 1.06 (0.92 - 1.22) | 0.74 (0.56 - 0.96)* | 0.81 (0.62 - 1.06) | 0.95 (0.74 - 1.22) | 1.04 (0.77 - 1.40) | 0.91 (0.61 - 1.36) | 0.96 (0.64 - 1.44) |  |
|  | 2022 | 1.28 (1.10 - 1.48)* | 1.41 (1.21 - 1.64)* | 1.05 (0.77 - 1.42) | 1.16 (0.86 - 1.56) | 1.07 (0.82 - 1.40) | 1.22 (0.91 - 1.64) | 1.76 (1.23 - 2.54)* | 1.75 (1.26 - 2.45)* |  |
| **QCDR measures (ACR)** | | **ACR14: Gout SU target  (N practice = 118)** | | **ACR10: HBV safety screening  (N practice = 116)** | | **ACR15: Safe HCQ dosing  (N practice = 113)** | | **ACR12: PsA disease activity measurement (N practice = 65)** | |  |
|  |  |  |  |  |  |  |  |  |  |  |
|  |  |  |  |  |  |  |  |  |  |  |
| **Characteristics** | | Unadjusted | Adjusted | Unadjusted | Adjusted | Unadjusted | Adjusted | Unadjusted | Adjusted |  |
|  |  | ORs (95% CI) | ORs (95% CI) | ORs (95% CI) | ORs (95% CI) | ORs (95% CI) | ORs (95% CI) | ORs (95% CI) | ORs (95% CI) |  |
| **Global dashboard engagement** | | | | | | | | | |  |
|  | None | REF | REF | REF | REF | REF | REF | REF | REF |  |
|  | Minimal | 1.27 (0.82 - 1.97) | 1.21 (0.82 - 1.77) | 1.15 (0.59 - 2.22) | 1.11 (0.63 - 1.96) | 0.79 (0.41 - 1.50) | 1.03 (0.53 - 1.99) | 0.44 (0.09 - 2.17) | 0.28 (0.08 - 0.98)* |  |
|  | Moderate | 1.17 (0.79 - 1.72) | 1.22 (0.87 - 1.71) | 1.03 (0.57 - 1.88) | 0.91 (0.55 - 1.51) | 0.97 (0.57 - 1.65) | 1.03 (0.59 - 1.80) | 0.61 (0.11 - 3.44) | 0.39 (0.09 - 1.72) |  |
|  | Most | 1.46 (0.96 - 2.23) | 1.47 (1.03 - 2.09)* | 1.55 (0.86 - 2.77) | 1.39 (0.81 - 2.39) | 0.82 (0.35 - 1.95) | 0.99 (0.50 - 1.98) | 0.77 (0.13 - 4.69) | 0.76 (0.16 - 3.70) |  |
| **EHR vendor** | | | | | | | | | |  |
|  | Nextgen | REF | REF | REF | REF | REF | REF | REF | REF |  |
|  | Amazing Charts | 1.18 (0.71 - 1.94) | 1.26 (0.78 - 2.05) | 0.90 (0.53 - 1.52) | 0.90 (0.52 - 1.55) | 0.52 (0.31 - 0.87)* | 0.48 (0.27 - 0.84)* | 0.54 (0.16 - 1.76) | 0.83 (0.35 - 1.99) |  |
|  | eClinicalWorks | 1.56 (1.15 - 2.11)* | 1.55 (1.14 - 2.11)* | 0.54 (0.35 - 0.82)* | 0.52 (0.33 - 0.81)* | 0.47 (0.21 - 1.08) | 0.48 (0.21 - 1.12) | 1.26 (0.52 - 3.09) | 1.15 (0.46 - 2.86) |  |
|  | eMDS | 0.87 (0.60 - 1.25) | 0.89 (0.62 - 1.26) | 0.93 (0.40 - 2.13) | 0.80 (0.34 - 1.85) | 1.23 (0.95 - 1.61) | 1.26 (0.93 - 1.72) | 1.88 (0.72 - 4.95) | 1.73 (0.75 - 3.98) |  |
|  | other | 1.06 (0.82 - 1.37) | 1.12 (0.90 - 1.41) | 0.58 (0.35 - 0.98)* | 0.51 (0.30 - 0.86)* | 1.05 (0.61 - 1.81) | 1.04 (0.60 - 1.78) | 0.59 (0.27 - 1.29) | 0.66 (0.31 - 1.41) |  |
| **Year** | | | | | | | | | |  |
|  | 2020 | REF | REF | REF | REF | REF | REF | REF | REF |  |
|  | 2021 | 0.97 (0.84 - 1.13) | 1.07 (0.93 - 1.24) | 0.66 (0.49 - 0.89)* | 0.67 (0.48 - 0.93)* | 0.65 (0.38 - 1.12) | 0.65 (0.38 - 1.12) | 1.82 (1.22 - 2.73)* | 2.48 (1.69 - 3.63)* |  |
|  | 2022 | 1.11 (0.90 - 1.37) | 1.31 (1.10 - 1.56)* | 1.38 (1.01 - 1.87)* | 1.44 (1.06 - 1.96)* | 0.62 (0.34 - 1.13) | 0.63 (0.36 - 1.11) | 2.50 (1.62 - 3.86)* | 3.82 (2.40 - 6.07)* |  |

^a^All models were binomial generalized linear models for practice-specific counts of patients meeting quality criteria (quality performance), with logit link function and denominators specified by the number of patients and adjusted for EHR vendor, year, and clustering by practice; global engagement was determined by practices' overall dashboard use.

**P* <.05.

**APPENDIX H. Sensitivity analysis, binomial generalized linear models: examining the association between any global engagement and quality performance^a^**

| **MIPS clinical quality measures (QPP)** | | **QPP39: Osteoporosis screening  (N practice = 164)** | **QPP178: RA functional status assessment (N practice = 156)** | **QPP177: RA periodic assessment of disease activity (N practice = 147)** | **QPP176: TB safety screening  (N practice = 149)** |
| --- | --- | --- | --- | --- | --- |
| **Characteristics** |  | Adjusted | Adjusted | Adjusted | Adjusted |
|  |  | ORs (95% CI) | ORs (95% CI) | ORs (95% CI) | ORs (95% CI) |
| **Any global dashboard engagement** | |  |  |  |  |
|  | No | REF | REF | REF | REF |
|  | Yes | 1.47 (0.97 - 2.21) | 1.32 (0.62 - 2.83) | 2.91 (1.29 - 6.61)* | 0.92 (0.57 - 1.49) |
| **EHR vendor** |  |  |  |  |  |
|  | Nextgen | REF | REF | REF | REF |
|  | Amazing Charts | 0.81 (0.40 - 1.64) | 0.27 (0.06 - 1.14) | 1.51 (0.77 - 2.93) | 0.42 (0.18 - 0.95)* |
|  | eClinicalWorks | 1.56 (1.03 - 2.37)* | 1.11 (0.55 - 2.24) | 1.04 (0.50 - 2.20) | 1.26 (0.42 - 3.75) |
|  | eMDS | 1.34 (0.79 - 2.25) | 0.60 (0.30 - 1.22) | 0.86 (0.35 - 2.11) | 1.88 (0.65 - 5.45) |
|  | other | 0.80 (0.52 - 1.22) | 0.82 (0.39 - 1.73) | 0.78 (0.34 - 1.76) | 1.29 (0.54 - 3.08) |
| **Year** |  |  |  |  |  |
|  | 2020 | REF | REF | REF | REF |
|  | 2021 | 1.00 (0.87 - 1.14) | 0.74 (0.57 - 0.96)* | 0.96 (0.73 - 1.28) | 0.94 (0.64 - 1.39) |
|  | 2022 | 1.32 (1.15 - 1.53)* | 1.06 (0.79 - 1.43) | 1.11 (0.83 - 1.49) | 1.70 (1.23 - 2.34)* |
| **QCDR measures (ACR)** | | **ACR14: Gout SU target  (N practice = 118)** | **ACR10: HBV safety screening  (N practice = 116)** | **ACR15: Safe HCQ dosing  (N practice = 113)** | **ACR12: PsA disease activity measurement (N practice = 65)** |
| **Characteristics** |  | Adjusted | Adjusted | Adjusted | Adjusted |
|  |  | ORs (95% CI) | ORs (95% CI) | ORs (95% CI) | ORs (95% CI) |
| **Any global dashboard engagement** | |  |  |  |  |
|  | No | REF | REF | REF | REF |
|  | Yes | 1.28 (0.91 - 1.78) | 1.03 (0.64 - 1.67) | 1.02 (0.59 - 1.76) | 0.53 (0.12 - 2.29) |
| **EHR vendor** |  |  |  |  |  |
|  | Nextgen | REF | REF | REF | REF |
|  | Amazing Charts | 1.19 (0.73 - 1.95) | 0.88 (0.53 - 1.47) | 0.48 (0.30 - 0.79)* | 0.61 (0.25 - 1.48) |
|  | eClinicalWorks | 1.61 (1.16 - 2.22)* | 0.54 (0.34 - 0.84)* | 0.48 (0.21 - 1.11) | 1.41 (0.55 - 3.58) |
|  | eMDS | 0.88 (0.61 - 1.25) | 0.87 (0.36 - 2.13) | 1.27 (0.98 - 1.65) | 1.95 (0.80 - 4.76) |
|  | other | 1.13 (0.90 - 1.42) | 0.47 (0.28 - 0.80)* | 1.05 (0.63 - 1.75) | 0.64 (0.31 - 1.33) |
| **Year** |  |  |  |  |  |
|  | 2020 | REF | REF | REF | REF |
|  | 2021 | 1.03 (0.90 - 1.19) | 0.62 (0.45 - 0.86)* | 0.65 (0.38 - 1.13) | 1.88 (1.27 - 2.79)* |
|  | 2022 | 1.25 (1.04 - 1.50)* | 1.36 (1.00 - 1.85)* | 0.64 (0.37 - 1.10) | 2.59 (1.68 - 4.00)* |

^a^All models were binomial generalized linear models for practice-specific counts of patients meeting quality criteria (quality performance), with logit link function and denominators specified by the number of patients and adjusted for EHR vendor, year, and clustering by practice; global engagement was determined by practices' overall dashboard use.

**P* <.05
